# Study protocol on the impact of postnatal maternal separation stress on dental enamel formation in a murine experimental model

**DOI:** 10.1371/journal.pone.0315667

**Published:** 2024-12-19

**Authors:** Júlia Ingryd Targino de Sousa, Juliana de Lima Gonçalves, Alexandra Mussolino de Queiroz, Fabrício Kitazono de Carvalho, Francisco Wanderley Garcia de Paula-Silva

**Affiliations:** Department of Pediatric Dentistry, Ribeirão Preto School of Dentistry, University of São Paulo, Ribeirão Preto, São Paulo, Brazil; Sapienza University of Rome: Universita degli Studi di Roma La Sapienza, ITALY

## Abstract

Dental development is a complex process influenced by genetic and environmental factors. Dental enamel, primarily composed of hydroxyapatite, is formed through complex cellular and biochemical mechanisms. Although this is a stable process, genetic, nutritional, and environmental factores can lead to developmental defects such as hypomineralization and hypoplasia. Molar incisor hypomineralization is a type of hypomineralization that represents a public health challenge. Its etiology is not yet fully understood, but factors such as hypoxia, medication exposure, adverse events in early childhood, and genetic influences are considered. This study protocol aims to investigate whether postnatal adverse events can impact amelogenesis, exploring the role of stress in the etiology of dental enamel defects. Specific objectives include evaluating enamel structure and mechanical properties by comparing the offspring of rats exposed to postnatal maternal separation with control animals (non-exposed). Additionally, we will evaluate weight, length, survival assessment, and developmental milestones between the groups. Macrophotographic analysis, microtomography, microhardness testing, and electron microscopy will enable a detailed assessment of enamel morphology and its mechanical properties. Histological and molecular analyses—such as immunohistochemistry, indirect immunofluorescence, and in situ zymography—will be performed to evaluate possible changes in proteins and enzymes that are essential for proper enamel biomineralization.

## Introduction

Dental development is a comprehensive and multifaceted process that begins through highly specialized cellular and biochemical mechanisms and is influenced by the interaction of genetic and environmental factors [1]. Enamel, the outer layer of the tooth, is a mineralized structure primarily composed of hydroxyapatite, a mineral abundant in calcium and phosphate ions [2]. This composition lends stiffness to its matrix, which exhibits a remarkable pattern of biomineralization [3].

Once dental enamel is formed, it remains unchanged, unlike other mineralized tissues [4]. However, despite this stability, various endogenous factors such as genetic predisposition and nutritional habits, as well as exogenous factors including excessive fluoride exposure, smoking, and alcohol consumption, can affect ameloblastic function [5]. These factors may result in anomalies in the enamel layer, leading to developmental defects of enamel (DDE). Some of the primary types of DDE include opacities, hypoplasia, and hypomineralization [6].

In the initial phase of dental enamel production, the secretion of matrix proteins plays a crucial role in enamel structure. Key proteins involved in amelogenesis, such as amelogenin (AMELX), ameloblastin (AMBN), and enamelin (ENAM), play essential roles in amelogenesis and subsequent biomineralization [7, 8]. It is crucial to emphasize that environmental interferences during this initial phase can lead to a reduction in enamel thickness, resulting in quantitative defects such as enamel hypoplasia [9]. Additionally, during the mineralization phase, hydroxyapatite crystals grow in volume and strengthen the enamel. If this phase is interrupted, the enamel layer becomes significantly more fragile, characterizing a qualitative defect known as enamel hypomineralization [10].

Molar incisor hypomineralization (MIH) is characterized as a qualitative defect in enamel layer development [11]. The etiology of MIH is not yet fully understood, but it tends to affect the first permanent molars and occasionally the incisors [12]. Global statistics on MIH indicate a prevalence ranging from 15.8% to 19.4% [13].

Various potential etiologies of MIH have been speculated in studies, including respiratory diseases, high fever, medication use, and exposure to toxic environmental substances such as bisphenol-A (BPA) and phthalates during the first three years of life [14–16]. Maternal stress, preterm birth, and complications during childbirth resulting in hypoxia can also impact enamel development [17–20]. This is due to the fact that the biomineralization process of deciduous molars and incisors begins in the later stages of pregnancy, while the process for permanent molars and incisors extends into the first three years of life [21].

Stress is a factor that can significantly impact dental mineralization. A recent study explored the relationship between stress, maturation, and dental development, using Linear Enamel Hypoplasia (LEH) monkey as an indicator of stressful influences during tooth formation. LEH manifests as horizontal lines or grooves in dental enamel, indicating disruptions in enamel formation due to stressful events during development. An interesting finding was that, during the prolonged childhood of these primates, the young are heavily dependent on their mothers. During this time, the process of tooth formation is closely related to overall growth and development, resulting in a greater likelihood of being affected by environmental, biological, and social stresses. For instance, changes in the environment, such as food scarcity or climate shifts, can cause significant stress. Additionally, diseases and infections can interfere with dental growth and development. Social factors, such as competition for resources and group dynamics, can also contribute to elevated stress levels [22].

Another study emphasized the comparison of estimated ages of formation of LEHs. It concluded that LEHs are treated as general indicators of metabolic stress, in addition to being a specific type of enamel thickness deficiency, particularly when occurring in early formation. This suggests that the presence of enamel alterations may indicate that the individual was subjected to metabolic stress during the period when the teeth were developing [23]. It is also crucial to recognize the influence of genes in amelogenesis, such as variations resulting from single nucleotide polymorphisms in the genes AMELX, MMP20, ENAM, and KLK4, which are associated with disorders in dental enamel development [24]. A notable example is amelogenesis imperfecta, considered the most severe form of enamel development alteration, resulting in fragile teeth that are susceptible to fractures [25].

Adverse events in early life have been linked to a poor response to maladaptive stress in adulthood [26, 27]. Studies show that various biological markers can identify exposure to these adverse events, including the presence of cortisol in hair strands and pronounced neonatal lines in deciduous teeth enamel [28, 29]. Recently, a cohort study revealed a link between stressful events, such as depression and anxiety during the pre- and perinatal periods, and the manifestation of more prominent neonatal lines in the deciduous teeth of exposed children compared to those who did not face such stressful situations. Although the biological mechanisms by which these factors influence dental enamel formation have not been fully unraveled, dental enamel is widely recognized as a sensitive indicator of physiological and pathological events. This is because a variety of influences—whether local, systemic, environmental, or genetic—can leave imprints in enamel development. Given that the etiology of developmental defects is associated with exposure to adverse events in early childhood, investigating the pathophysiological mechanisms underlying the influence of toxic stress on enamel biomineralization becomes relevant [30].

Stress is a physiological response of the body, and when it occurs in isolated events, it does not have negative impacts on health; moreover, it is essential for human survival. Stress can be classified as positive stress, which is an isolated situation such as learning a new skill; tolerable stress, which is frequent stressful situations, such as the loss of a family member for children; and toxic stress, which is an intense and continuous presence of stressful events, such as parental neglect, physical and verbal abuse, socioeconomic problems, and bullying [31]. Toxic stress can impact child development and growth, and a recent study pointed out that adverse events during early life can also affect enamel formation [32].

It is important to emphasize that stressful factors in the environment during the early postnatal period can trigger irreversible neuropsychiatric conditions in adulthood. An important component is the hypothalamic-pituitary-adrenal (HPA) axis, which plays a fundamental role in responding to adverse stress [33]. The HPA axis is activated by both internal and external stimuli, resulting in increased release of hormones such as corticosterone and adrenocorticotropic hormone, which play important roles in regulating steroid production by the adrenal cortex [34, 35]. These processes may relate to negative impacts and contribute to the manifestation of aversive memories and the development of depression [36]. Maternal separation, often used for behavioral analyses, elevates levels of corticosterone and corticotrophin-releasing hormone released by the hypothalamus [37]. The maternal separation protocol in rodents can replicate the impacts observed in humans after adverse experiences in the postnatal period. Repeated early maternal separation, combined with a physical stressor (cold stress), resulted in offspring showing an increase in voluntary alcohol consumption and plasma corticosterone levels [38].

This study aims to investigate whether postnatal maternal separation stress impacts enamel development in a murine experimental model. Analysis will be performed on structural features, mechanical properties of the enamel layer, and molecular components from amelogenesis. Additionally, this study will evaluate whether the developmental milestones of rat offspring will be affected due to exposure to adverse events. The findings of this study will help explore how stress may potentially be linked to the etiology of MIH, providing a more comprehensive understanding of the underlying factors of this specific dental condition.

## Materials and methods

### Animal procedures

For this experiment, four pregnant Wistar rats (*Rattus norvegicus*, *albinus*) with a gestation period of three weeks, weighing approximately 300 g, will be obtained from the Central Animal Facility at USP Ribeirão Preto Campus. The project was reviewed and approved by the Ethics Committee on Animal Use at FORP/USP prior to experimentation (Protocol number 0103/2024R1). All experimental procedures will be conducted following the guidelines of the National Council for Control of Animal Experimentation (CONCEA) and the ARRIVE guidelines. The animals will be housed in the Animal Facility at the School of Dentistry of Ribeirão Preto—USP, in polypropylene cages with perforated stainless-steel lids, measuring 15 × 20 cm, lined with wood shavings. They will be maintained at a constant temperature of 22°C and relative humidity of 55 ± 10%, under a 12:12 light-dark cycle throughout the experimental period, with a standard laboratory diet and free access to filtered waterFor the induction of stress in the animals, a model of offspring separation from the mother will be used to increase stress levels in rat offspring. For this purpose, the following groups will be constituted: maternal separation group (n = 20) and control group (n = 20).

Maternal separation will begin on the second day of life for the offspring (T2). They will be removed from the nest for a period of 4 hours per day for 28 days and kept in an incubator at 32°C. Subsequently, the animals will be returned to the nest with their mothers. The animals in the control group will remain with their mothers in the nest, and no intervention will be performed [39].

**Fig 1:**
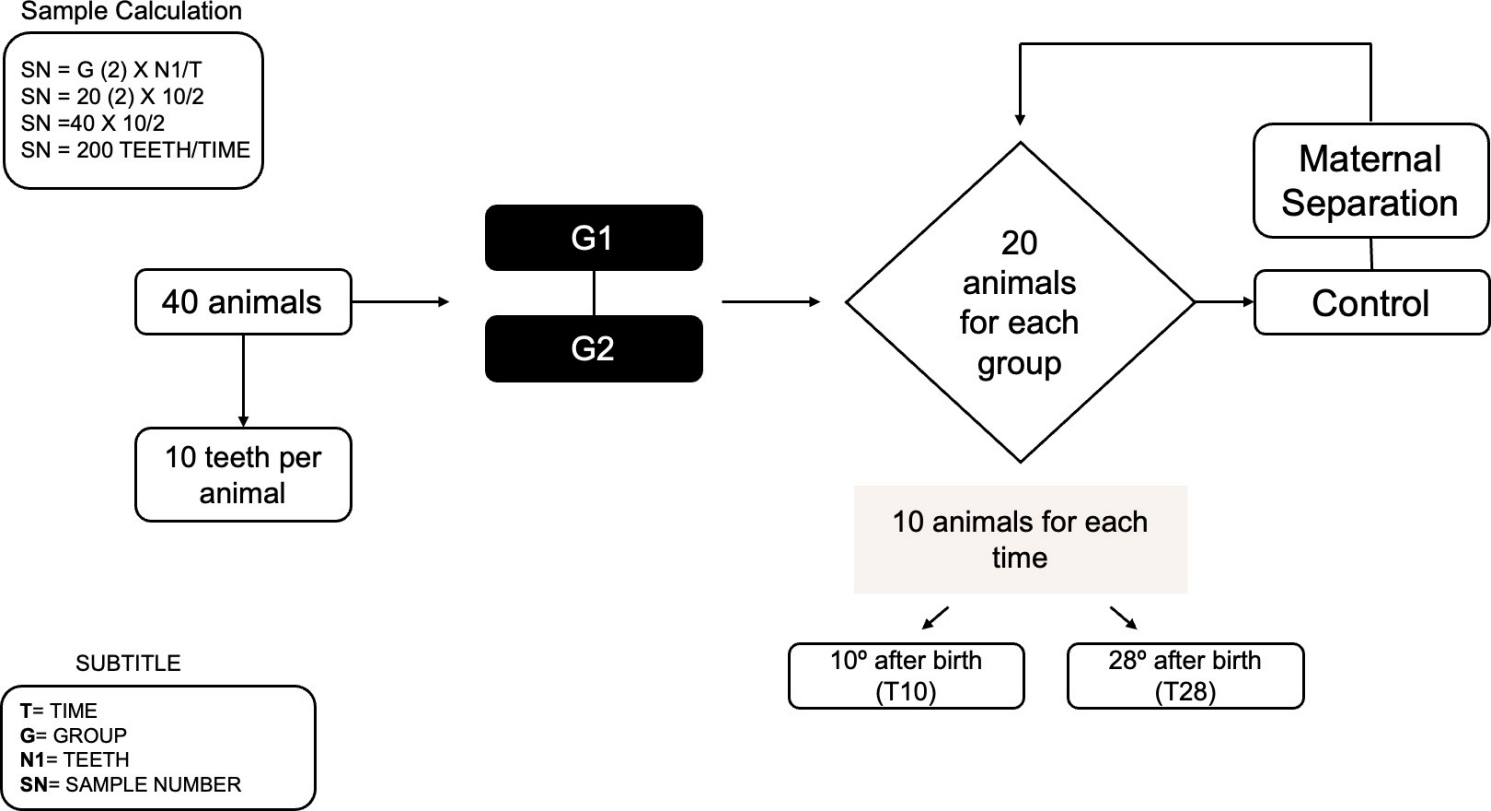
Design for obtaining the sample number. This scheme represents the experimental design for specimen collection, involving a total of 40 animals distributed into two groups, with 20 animals per group. The groups are as follows: Group 1—Maternal Separation (20 animals) and Group 2—Control (20 animals). The experiment will consist of two phases. On the 10th day after birth (T10), a group of animals will be euthanized (n = 10 animals per group) for the collection of tissues containing incisors and first molar germs. On the 28th day after birth (T28), the other group of animals will be euthanized (n = 10 animals per group) for the collection of incisors and first molars that have already erupted in the oral cavity.

On the 10th day after birth (T10), euthanasia will be performed on half of the animals in the control and experimental groups (n = 10 animals per group) for tissue collection containing the incisors and first molar germs. On the 28th day after birth, euthanasia will be performed on the other half of the animals from the control and experimental groups (T28; n = 10 animals per group) for the collection of incisors and first molars that have already erupted in the oral cavity (Fig 1). The analyses to be performed will be comparative, using samples obtained from male and female animals to investigate possible differences between sexes. Rat molars and incisors have distinct characteristics during development. The incisors exhibit continuous growth, while molars do not have this characteristic. From each animal, two incisors and two molars will be collected, resulting in a total of 40 teeth per group. Previous studies from our group have shown that microhardness analysis requires more samples due to variable results, so 10 teeth per group will be needed, as the sample size calculation was based on prior studies [40, 41]. For each group, twenty teeth will be used for microhardness analysis; after the indentations, eight teeth will be allocated for SEM and EDS analysis, and another eight for Raman spectroscopy. For microCT analysis, eight teeth will be used. To obtain histological slides, six hemi-mandibles containing the molars and incisors will be used, followed by hematoxylin and eosin staining, immunohistochemistry, indirect immunofluorescence, and *in situ* zymography (α = 5%; β = 0.2; test power 80%).

### Euthanasia of the animals

The animals will be anesthetized using a combination of ketamine (100 mg/kg; Agener®, Agener União, São Paulo, SP, Brazil) and xylazine (7.5 mg/kg; Syntec®, Syntec, Santana de Parnaíba, SP, Brazil) administered intramuscularly. Following anesthesia, the animals will be placed in a carbon dioxide chamber calibrated to a flow rate of 7 L/min. Subsequently, the lower and upper incisors, along with tissues containing bone and teeth, will be collected for further analysis.

### Systemic analysis

#### Evaluation of body weight and length

Animals and any possible alterations between the control and experimental groups. After birth, the animals will be weighed at birth and on the 7th, 10th, 14th, 21st, and 28th days. In addition to weight, the offspring will be measured at birth and on the 7th, 10th, 14th, 21st, and 28th days to assess body length (from head to tail).

#### Survival assessment

The assessment will be based on the survival curve using the Kaplan-Meier estimator. The objective is to evaluate the animals from the first to the last day of the experimental period, thereby estimating the survival rate and mortality of these animals due to the environmental exposures they experienced [42].

#### Assessment of physical development milestones in animals

During the experimental period, the development of the animals will be monitored, and comparisons will be made between the control and experimental groups. The aim is to identify potential developmental alterations resulting from exposure to adverse events related to maternal separation. The day of incisor eruption will be recorded by examining the oral cavity of the animals daily until the tip of the lower incisor erupts through the mucosa. Additionally, other parameters of physical development, such as the timing of ear and eye opening, the initiation of solid food intake, and the weaning period, will be observed [43].

#### Evaluation of plasma levels of cortisone and ACTH

This technique will be performed on the day of euthanasia, with the rat anesthetized and positioned in dorsal recumbency, ensuring that its forelimbs are extended. The thorax of the animal will be swabbed with 70% ethyl alcohol, and a needle will be inserted on the left side of the xiphoid cartilage, near the base of the sternum, forming an angle of 20 to 30 degrees relative to the body of the animal. The needle will be inserted slowly while applying slight negative pressure to the syringe plunger. Blood should flow into the needle hub as soon as the bevel penetrates one of the chambers of the heart. Subsequently, the syringe plunger will be pulled continuously and slowly until blood flow is complete [44].

### Molecular analysis of dental germs (incisors and first molars)

#### Histotechnical processing

After euthanasia, the mandibles will be dissected, and the tissues will be fixed in 10% buffered formalin for 24 hours at room temperature. They will then be demineralized in 5% ethylenediaminetetraacetic acid (EDTA; pH 7.4) for approximately 21 days. Following demineralization, the specimens will undergo routine histotechnical processing, which includes washing in running water for 24 hours, dehydration in increasing concentrations of alcohol, clearing in xylene, and embedding in paraffin. The blocks containing teeth and bone tissue will be longitudinally sectioned on a microtome (Leica RM2145, Leica Microsystems, Wetzlar, Germany) into 5 μm-thick sequential sections. The resulting slides will be stained with hematoxylin and eosin for microscopic evaluation, as well as for immunochemistry, indirect immunofluorescence, and *in situ* zymography.

#### Microscopic analysis of dental enamel formation

The analysis of dental enamel formation (including the organic matrix and ameloblasts) will be conducted on hematoxylin and eosin-stained slides using a Zeiss Axiolab 5 microscope (Zeiss, Göttingen, Germany) under bright-field illumination. The morphological aspects of ameloblasts, such as shape and nuclear polarization, will be evaluated in comparison to the control group. Additionally, cells from amelogenesis and the junctions between ameloblasts will be analyzed.

#### Immunohistochemistry

Immunohistochemistry will be performed to detect the synthesis and distribution of proteins along the ameloblast layer, enabling an analysis of potential alterations in the synthesis of the investigated proteins. Antibodies targeting cell junction proteins (claudins), amelogenesis proteins (amelogenin, ameloblastin, tuftelin, enamelin), enzymes (KLK-4, MMP-20, COX-2), and albumin (serum and fetal) will be utilized.

Slides will be deparaffinized and hydrated through a decreasing series of ethanol, then maintained in phosphate-buffered saline (PBS). Following this, tissue sections will be microwaved (7 × 12 seconds at 2-minute intervals) in sodium citrate buffer (pH 6.0) for antigen retrieval. After temperature stabilization, the slides will be washed with PBS (three times for 5 minutes), and endogenous peroxidase activity will be blocked using 3% hydrogen peroxide for 40 minutes. Subsequently, the slides will be washed again with PBS (three times for 5 minutes), and non-specific binding sites will be blocked with 5% bovine serum albumin (Sigma-Aldrich) for 60 minutes. Tissues will be incubated with the primary antibodies mentioned above at 4°C overnight. The following day, slides will be washed and incubated with a biotinylated secondary antibody for 1 hour, then washed in PBS before being incubated with streptavidin conjugated to horseradish peroxidase (HRP) for 20 minutes. For visualization, 3,3′-diaminobenzidine (DAB, Sigma-Aldrich) will be used as the enzyme substrate for 5 minutes; after washing with PBS, the slides will be counterstained with hematoxylin for 15 seconds, washed with distilled water, dehydrated through increasing concentrations of ethanol, and mounted in Entellan® (Merck, Darmstadt, Germany). Negative control slides, in which the primary antibody is omitted, will be included to assess the specificity of the immunostaining. Quantification of the intensity of immunostaining in the ameloblast layer and the enamel organic matrix will be performed using ImageJ software (National Institutes of Health, Bethesda, MD, USA) along with the image deconvolution plugin (Color Deconvolution). DAB vector will be applied, and the selected channel and threshold will be manually adjusted at 20× magnification.

#### Indirect immunofluorescence

The transcription factor Runx2 plays a crucial role in the differentiation of osteoblasts, odontoblasts, and ameloblasts. Specifically, in enamel, Runx2 deficiency affects the production of the enzyme KLK4 and amelogenins, leading to impaired mineralization of the enamel matrix. To investigate the synthesis and nuclear translocation of Runx2 and its role in biomineralization, indirect immunofluorescence will be conducted as previously described [45].

Slides will be prepared as outlined in the immunochemistry section. Following this, the slides will be washed with PBS (three times for 5 minutes) and treated with a 1 mg/mL sodium borohydride solution (three times) (Dinâmica Química Contemporânea Ltda., Diadema, SP, Brazil) for 15 minutes. Non-specific binding sites will be blocked with 5% bovine serum albumin (Sigma-Aldrich) for 60 minutes. Immunolabeling will be performed using a primary antibody against runt-related transcription factor 2, incubated at 4°C overnight. The following day, the slides will be washed in PBS (three times) and incubated with a secondary antibody (mouse anti-rabbit IgG conjugated with fluorescein) for 1 hour in a dark chamber. After washing the slides with PBS (three times), the nuclei will be stained with 4′,6-diamidino-2-phenylindole (DAPI) (0.5 μg/mL) (Santa Cruz Biotechnology Inc., Dallas, TX, USA) for 5 minutes. The slides will then be mounted with ProLong Gold Antifade (Molecular Probes Inc., Eugene, USA). Images will be captured using fluorescence microscopy at 20× magnification with fluorescein isothiocyanate (FITC) and DAPI filters. The resulting images will be analyzed using ImageJ software (U.S. National Institutes of Health, Bethesda, MD, USA). The channels will be separated, and using the “green” channel, the ameloblast layer will be delineated and measured. The ratio values for “integrated density” and area will be calculated.

#### *In situ* zymography

Matrix metalloproteinase-20 (MMP-20) is an enzyme that plays a crucial role in the removal of organic content during the mineralization phase of the enamel matrix. By facilitating the elimination of organic material, MMP-20 allows hydroxyapatite crystals to expand and incorporate minerals, thereby ensuring that enamel retains its essential characteristics, such as high hardness.

In situ zymography will be performed to detect the proteolytic activity of matrix metalloproteinases in the ameloblast layer and the organic matrix of enamel. When combined with immunohistochemical analysis, this technique will enable the identification of the enzyme. Slides will be prepared as outlined in the immunochemistry section and subsequently immersed in a sodium borohydride solution (1 mg/mL) for 15 minutes (three times). The tissues will then be incubated with a fluorescein isothiocyanate (FITC)-bound gelatin substrate (DQ™ Gelatin, Molecular Probes, Eugene, OR) dissolved in agarose (0.1 mg/mL) for 2 hours at 37°C in a humidified, light-protected chamber. Nuclei will be counterstained with 4′,6-diamidino-2-phenylindole (DAPI; 0.5 μg/mL) added to the incubation medium. As a control, slides will be preincubated in 20 mM ethylenediaminetetraacetic acid (EDTA, Sigma, St. Louis, MO) for 1 hour, after which EDTA will also be added to the incubation medium. Quantification of gelatinolytic activity will be conducted using ImageJ software. Images captured with the FITC filter at 20× magnification will be analyzed. After separating the channels, the green channel will be selected to delineate the ameloblast layer, and the integrated density value and area ratio will be calculated.

### Quantitative and qualitative analysis of dental enamel

#### Macrophotographic analysis

To analyze the presence of clinically visible alterations in dental structures, photographs will be taken after the euthanasia of the animals using a digital camera (Eos Rebel T2i, Canon, Tokyo, Japan) equipped with a 100 mm f/2.8 macro lens (Canon, Tokyo, Japan). The camera settings will be ISO 200, f/32, and a 3-second exposure time. To enhance the visualization of the enamel surface, polarized and infrared light filters will be utilized. Photographs will be captured of the vestibular surfaces of the incisors and the occlusal surfaces of both upper and lower molars [46].

#### Microcomputed tomography

Microcomputed tomography (microCT) analysis of the enamel layer size and volume will be conducted using the Phoenix V|Tome|X S microCT scanner (General Electric, Boston, USA) at the Biodiversity Documentation Center (CDB), Department of Biology, Faculty of Philosophy, Sciences and Letters of Ribeirão Preto (FFCLRP/USP). First molars and lower incisors will be collected from both the control and experimental groups. After cleaning and drying, each tooth will be placed in microtubes and positioned perpendicular to the radiation source. Scanning parameters will be set at 70 kV and 200 μA, with a 0.1 mm Al/Cu filter and a voxel size of 5.4 μm. The images will then be reconstructed using 3D slice software [47], and analyses will be performed using ImageJ software (Wayne Rasband, National Institutes of Health, USA). Hydroxyapatite (Ca₁₀(PO₄)₆(OH)₂) will be used for standardization and calibration of measurements. Enamel volume, thickness, and density will be measured from the microcomputed tomography sections.

#### Knoop microhardness test

The microhardness test will be conducted to assess changes in the enamel resistance of incisors in both the control and maternal separation groups. Samples will be dried and embedded in acrylic resin. The block containing the tooth will then be cut into sagittal sections, ground, and polished before being air-dried for 24 hours. The samples will be taken to the microhardness tester (Shimadzu–HMV-2, Kyoto, Japan) at the Multiuser Center for Biomechanical Studies in Dentistry (CMEBiO), Department of Dental Materials and Prosthodontics, Faculty of Dentistry of Ribeirão Preto, University of São Paulo. A Knoop diamond tip will be used with a load of 10 gf for 5 seconds. Three indentations will be made in three different regions of each sample: the tip, middle part, and cervical portion, and the average hardness of each tooth will be calculated.

#### Scanning electron microscopy

Scanning electron microscopy (SEM) is a method that provides high-resolution images of surfaces. It will be utilized to analyze the enamel surface and prisms, as well as any morphological changes present. For the images of the enamel surface of incisors, the teeth will be air-dried for 24 hours before being placed in the scanning electron microscope (JEOL-JSM-6610LV). Images will be captured at magnifications of 500x and 2000x.

To obtain images of enamel structure, molars and incisors extracted from the animals will undergo surface treatment. The teeth will be air-dried and embedded in acrylic resin. The blocks containing the teeth will be cut into cross-sectional sections. A 37% phosphoric acid solution will be applied for 30 seconds, followed by washing, sonication, and air drying for 24 hours. Once the surface preparation is complete, a layer of carbon will be applied to enhance visualization. The samples will then be fixed in the electron microscopy device (JEOL-JSM-6610LV), and images will be acquired at magnifications of 150x, 750x, and 3000x. For sagittal sections, the blocks will be ground using a polishing machine, gradually decreasing the grit of the abrasive paper (#400, #800, and #1200). The same surface treatment protocol described above will then be repeated.

#### Energy-dispersive X-ray spectroscopy

For the quantification of mineral content and potential alterations, energy-dispersive X-ray spectroscopy (EDS) analysis will be performed using the Oxford Instruments INCA 300 EDX System (Abingdon, Oxfordshire, United Kingdom) at the Multiuser Equipment and Services Center of the Faculty of Medicine of Ribeirão Preto (FMRP/USP). The elemental content will be expressed as the percentage of the total weight of each element relative to 100%. Counting will be conducted in both the incisal/occlusal and cervical regions of the incisors and molars. The quantified elements will include calcium (Ca), phosphorus (P), oxygen (O), and carbon (C).

#### RT-Raman

Raman spectroscopy analysis is a scattering technique in which a laser beam is directed onto a sample, causing the frequency of the scattered radiation to be either higher or lower than that of the incident radiation. This technique is used to detect chemical components and their quantities within each sample. In this experiment, Raman spectroscopy will be employed to investigate the amounts of organic and mineral components in the teeth of rats and to identify any alterations in these components within the enamel. The laser will be focused on the labial and occlusal surfaces of each tooth using a ×50 objective at three points: the tip/occlusal, middle region, and cervical portion, with a laser power of 15 mW [48].

#### Statistical analysis

The obtained results will be analyzed using GraphPad Prism 10.0 software (Prism, Chicago, IL, USA). Data will be subjected to the Shapiro-Wilk normality test; if the data exhibit a normal distribution (p > 0.05), they will be analyzed using a T-test. If the data show a non-normal distribution (p < 0.05), the nonparametric Mann-Whitney U test will be applied. The significance level will be set at 5%.
